# Supplementary material for: Reversible Dimerization of Human Serum Albumin
Source: Molecules. 2020 Dec 29;26(1):108. doi: 10.3390/molecules26010108 (PMC7795135; doi:10.3390/molecules26010108)
Supplement: Supplementary file 1 [file molecules-26-00108-s001.pdf]

Reversible dimerization of human serum albumin

Alexey Chubarov [1,2,*], Anna Spitsyna [2,4], Olesya Krumkacheva [2,3,*], Dmitry Mitin [1,2], Daniil Suvorov [1,2], Victor Tormyshev [4], Matvey Fedin [2,3], Michael K. Bowman [4,5,*] and Elena Bagryanskaya [4,*]

[1] Institute of Chemical Biology and Fundamental Medicine, SB RAS 630090 Novosibirsk, Russia;
[2] Novosibirsk State University, 630090 Novosibirsk, Russia;
[3] International Tomography Center, SB RAS 630090 Novosibirsk, Russia;
[4] N.N. Vorozhtsov Institute of Organic Chemistry, SB RAS 630090 Novosibirsk, Russia;
[5] Department of Chemistry & Biochemistry, University of Alabama, Tuscaloosa, AL 35487, USA
* Correspondence: mkbowman@ua.edu (M.K.B.); chubarovalesha@mail.ru, chubarov@niboch.nsc.ru (A.C.); egbagryanskaya@nioch.nsc.ru (E.B.); olesya@tomo.nsc.ru (O.K.); Tel.: +7-383-330-8850 (E.B.); +7-913-763-1420 (A.C.)

Оглавление

1. MATERIALS AND METHODS 1
2. SYNTHESIS AND CHARACTERIZATION OF HSA-NIT AND HSA-OX063 CONJUGATES 2

Fractionation of Albumin on Sephadex G-150 2

Synthesis of HSA-NIT and HSA-OX063 conjugates 3

Gel electrophoresis (SDS-PAGE) and Circular dichroism (CD) data of albumin conjugates 4

Continuous wave EPR results 5

Preparation of the samples for DEER 7

DEER 8

Characterization of HSA conjugates Structures by Dynamic light scattering (DLS) 11
3. REFERENCES 13

1. Materials and methods

Materials. The human serum albumin (HSA) used in this study was purchased from Sigma–Aldrich Chem. Co. (A3782, USA). MS (MALDI ToF) m/z HSA 66.48 kDa. HSA has 35 Cys residues, with 34 paired in 17 disulfide bonds. The remaining unpaired Cys34 either is reduced, with a free SH group, (in healthy adults, about 70–80 % of the HSA) or is oxidized as a disulfide (20-25 %) or as a sulfinic or sulfonic acid (2-5 %). Ellman's test indicated that the starting HSA in this study contained 0.29±0.05 sulfhydryl groups per protein molecule. 5,5′-Dithio-bis(2-nitrobenzoic acid) (DTNB) and all solvents and other reagents were purchased from Sigma (St. Louis, MO) at the highest available grade and used without purification. Centricon concentrators with 3 kDa molecular weight cut-off were purchased from Millipore.

The number of thiol groups per albumin molecule was determined using Ellman's method as described in the literature [1] or on the Thermo Scientific website with 5,5′-Dithio-bis(2-nitrobenzoic acid) (DTNB) at pH 8 at 412 nm ($\varepsilon = 1.4 \times 10^4\ M^{-1}\ cm^{-1}$). DTNB produces a measurable yellow-colored product when it reacts with free SH groups.

Electronic absorption spectra were recorded on a UV-1800 spectrometer (Shimadzu, Japan). The concentrations of HSA solutions were determined by absorption at 278 nm, pH 7.4 (PBS), using a molar extinction coefficient $\varepsilon = 3.7\times10^4$ $M^{-1}cm^{-1}$.

CW EPR. Continuous-wave (CW) EPR was measured for HSA solutions in deuterated potassium PBS buffer. Samples were placed in glass capillary tubes (OD 1.5 mm, ID 0.9 mm). Continuous-wave (CW) EPR measurements were carried out at X-band at room temperature using a commercial X-band Bruker EMX spectrometer. Double integrals were calculated with Bruker WinEPR software. Spin concentration was determined by comparing spectral double integrals of the sample and a nitroxide solution with a known concentration.

Experimental CW EPR settings for nitroxide label were as follows: sweep width, 12 mT; microwave power, 6.315 μW; modulation frequency, 100 kHz; modulation amplitude, 0.1 mT; time constant, 20.48 ms; conversion time, 81.92 ms; the number of points, 1024; number of scans, 4. Experimental CW EPR settings for samples with OX063 label were as follows: sweep width, 2 mT; microwave power, 1.263 μW; modulation frequency, 100 kHz; modulation amplitude, 0.05 mT; time constant, 20.48 ms; conversion time, 81.92 ms; the number of points, 1024; number of scans, 2.

Circular dichroism (CD) data were collected at 25 °C with a JASCO J-600 spectrophotometer with the time constant 4 s, bandwidth 1 nm, using a 0.01 cm path length quartz cell. All CD spectra were obtained in triplicate from 190 to 240 nm, and the final spectrum was obtained as an average of 10 spectra. To determine the percentages of α-helices, β-sheets, and disordered structures, the difference between the theoretical and experimental curves was minimized. The theoretical curves were calculated as a linear combination of the basis spectra of various components of the secondary structures taken from the CCA+ software [2]. For recording the CD spectra, samples consisted of 5 μM albumin in PBS.

SDS-PAGE. Human serum albumin conjugates were analyzed by sodium dodecyl sulfate polyacrylamide gel electrophoresis using 7 % PAAG under Laemmli condition without the addition of dithiothreitol (DTT) with subsequent Coomassie Brilliant Blue staining. Quantitative data were obtained by digitizing the gel using GelPro Analyzer software.

2. Synthesis and characterization of HSA-NIT and HSA-OX063 conjugates

Fractionation of Albumin on Sephadex G-150

The fractionation procedure was adapted from Janatova et al. [1]. The Sephadex G-150 was suspended in 0.1 M KCl according to the standard manufacturing procedure. Then 1 ml of a 1 mM HSA solution in 0.1 M KCl was applied to the

Sephadex column. The solution of 0.1 M KCl was then passed through the column. The absorbance of the eluate was monitored with a UV- 1800 spectrometer (Shimadzu, Japan). Each eluate probe was analyzed by SDS-PAGE (Figure S1). The monomer and oligomer fractions were concentrated using Centricon concentrators with a 3 kDa molecular weight cut-off. The protein was reconstituted in PBS buffer, and the process was repeated not less than 4 times using PBS as eluent. To evaluate the differences in albumin secondary structure circular dichroism (CD) data of the initial albumin and monomer (m-HSA) and oligomer (o-HSA) fractions were analyzed. Changes in the secondary structure of the protein were examined by deconvolution of CD spectra to determine the α-helix and β-sheet content (Table S1). The secondary structure of the HSA remained intact: α-helical content of the m-HSA conjugates increased slightly from 54.9% to 56.4%, while the β-sheet content decreased from 3.7% to 2.6%. In contrast to m-HSA, the o-HSA fraction showed the opposite trend.

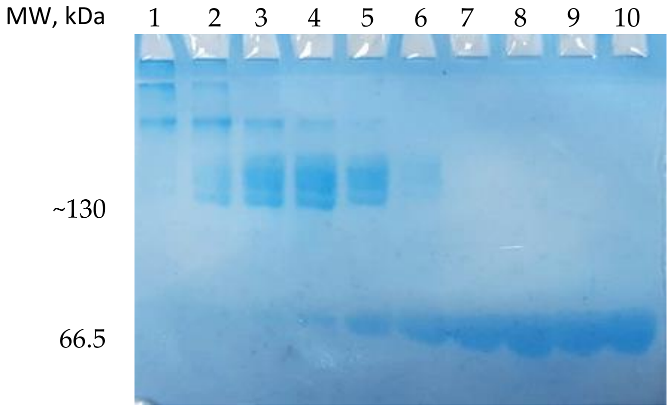

Figure S1. SDS-PAGE analysis of the fractionation of albumin on Sephadex G-150 under Laemmli condition without the addition of DTT with subsequent Coomassie blue staining. Lanes 1-6 contain an oligomer form of HSA. Lanes 3-4 contain the main part of the HSA oligomers (o-HSA). Lanes 7-10 contain the monomer form of HSA and will be known as the monomer fraction of HSA (m-HSA).

Synthesis of HSA-NIT and HSA-OX063 conjugates

The synthetic procedure for nitroxide labeled HSA-NIT was adapted from Krumkacheva et al. [3]. The synthetic procedure for OX063 labeled HSA-OX063 was adapted from Tormyshev et al. [4]. The methanethiosulfonate derivative of OX063 with an amide replacing the ester links was used for the albumin modification to obtain stable protein conjugates (Figure 1) [4].

Briefly, 0.1 mL (0.3 mM) solution of the monomer fraction of HSA (m-HSA) in PBS (10 mM sodium phosphates, 140 mM NaCl, 2.68 mM KCl, pH 7.4) was mixed with 5 μl 1.2-fold excess of DTT dissolved in PBS and stirred at 37 °C for 20 min. Unreacted low molecular weight materials were immediately removed by centrifugal filtration using Centricon

concentrators with a 3 kDa molecular weight cut-off using PBS as eluent. The reduction of HSA yielding HSA with free Cys-34 (mercaptalbumin or m-HSA-SH) was performed by DTT as described in the literature [5]. Under these conditions, no intramolecular disulfides (protein S-S bridges) were broken by DTT. Using 5,5′-Dithio-bis(2-nitrobenzoic acid) (DTNB) as described on the Thermo Scientific website it was found that m-HSA contains 0.9 ± 0.1 sulfhydryl group per protein molecule. The SDS-PAGE gel retardation assay data before and after the reduction is presented in Figure S1 and S2. No significant changes in m-HSA before or after the addition of DTT have been detected. CD data is presented in Table S1. HSA samples were used immediately for the next step of the synthesis.

300 μl (0.5 mM) of m-HSA solution in PBS was mixed with a 4-fold excess of methanethiosulfonate derivatives of nitroxide or OX063 radical dissolved in 18 μL DMSO and stirred at 37 °C overnight. Unreacted low molecular weight materials were removed using Centricon concentrators. Final samples for CW EPR and DEER experiments were prepared using Centricon concentrators and potassium PBS in D_2O (2 mM KH_2PO_4, 10 mM K_2HPO_4, 140 mM KCl pH 7.4) as eluent. The yield of spin-labeled HSA was ~ 95%. The incorporation of radical residues into HSA was proven by UV-vis., EPR, and Ellman's assay for free SH-groups, with unmodified HSA as control. UV–vis of m-HSA-NIT (PBS, pH 7.4) gave a peak at λ_{max}=278 nm. Analyses of CW EPR and UV spectra of m-HSA-NIT showed that ~97 % of the HSA is spin-labeled by nitroxide. UV–vis m-HSA-OX063 (PBS, pH 7.4) gave peaks at λ_{max} 278 nm, 465 nm. Analyses of CW EPR and UV spectra of m-HSA-OX063 showed that ~79 % of the HSA is spin-labeled by trityl.

Gel electrophoresis (SDS-PAGE) and Circular dichroism (CD) data of albumin conjugates

Any protein modification might cause protein damage, loss of function, conformational changes and precipitation, etc. Gel electrophoresis (SDS-PAGE) was used to check the HSA conjugates for any changes in mobility from that of HSA (Figure S2.) A band corresponding to monomeric protein (MW = 66.5 kDa) only was observed in m-HSA-NIT and m-HSA-OX063 including the starting m-HSA, but the m-HSA-OX063 conjugate showed ~ 1% of the dimer formation.

Changes in the secondary structure of the protein were examined by deconvolution of CD spectra to determine the α-helix and β-sheet content (Table S1). The secondary structure of the m-HSA remained intact: α-helical content of the m-HSA-NIT and m-HSA-OX063 conjugates decreased slightly from 56.4% to 53.1%-54.7%, while the β-sheet increased from 2.6% to 4.0%-4.8%. These slight changes show that albumin modification by spin labels doesn't cause significant changes in the HSA secondary structure. Albumin possesses seven major binding sites for fatty acids with high and

moderate affinity. To compare the secondary structure of HSA conjugates in conditions more similar to physiologic we added ~ 25 fold excess of the myristic acid to the m-HSA samples. The addition of the myristic acid increased the α-helical content by 2%-3% and slightly decreased the β-sheet content in all m-HSA samples.

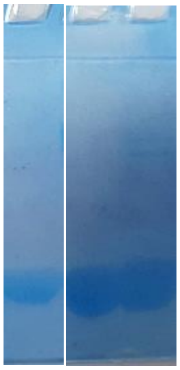

Figure S2. SDS–PAGE of albumin conjugates under Laemmli condition without addition of DTT with subsequent Coomassie blue staining; m-HSA-SH (lane 1); m-HSA-NIT (lane 2); m-HSA-OX063 (lane 3).

Table S1. HSA conjugates secondary structure from CD

HSA Type	α-Helix, %	β-Sheet, %	SH-groups[2]	Label, %
HSA	54.9	3.7	0.29±0.05	-
m-HSA	56.4	2.6	0.33±0.05	-
m-HSA-SH[1]	53.7	3.7	0.90±0.10	-
o-HSA	52.2	6.2	0.20±0.05	-
m-HSA-NIT	54.7	4.0	~ 0	97
m-HSA-OX063	53.1	4.8	0.10±0.05	79
m-HSA-SH + myr. acid	56.5	3.2	-	-
m-HSA-NIT + myr. acid	55.8	3.4	-	-
m-HSA-OX063 + myr. acid	55.2	3.5	-	-

[1] The sample was obtained by the reduction of HSA by DTT.

[2]SH-groups per albumin was determined by Ellman's test

Continuous wave EPR results

Continuous wave (CW) EPR spectra contain information on the mobility, the local environment of the spin label and its interactions with other spins. Nitroxide spectrum shape strongly depends on the label mobility due to hyperfine interaction with ^{14}N nuclear spin leading to appearance of three lines in the spectrum. The m-HSA-NIT spectrum indicates slow rotational motion of the spin label, Figure S3, A. This spectrum contains two strongly-overlapped components with different mobility and rotational correlation times ~2 and ~10 ns and small amount (< 5%) of free spin label [6]. Presumably, the two components in the spectrum represent two types of conformations for the MTSL conjugated to Cys-34. The amount of free spin label varies slightly from sample to sample because each sample was

cleaned and centrifuged separately, Figure S4, but the minute mount of free lable does not interefere with the DEER measurements. Analogous spectra were measured for m-HSA-OX063 with the trityl spin label. The trityl spectrum has little dependence on label mobility because it has a single, featureless, narrow line, Figure S3, B. Therefore, all m-HSA-OX063 CW EPR spectra are similar, Figure S5, except for a slight broadening for m-HSA-OX063 mixed with myristic acid. None of the CW EPR spectra show major broadening indicative of two spin labels on the same HSA monomer.

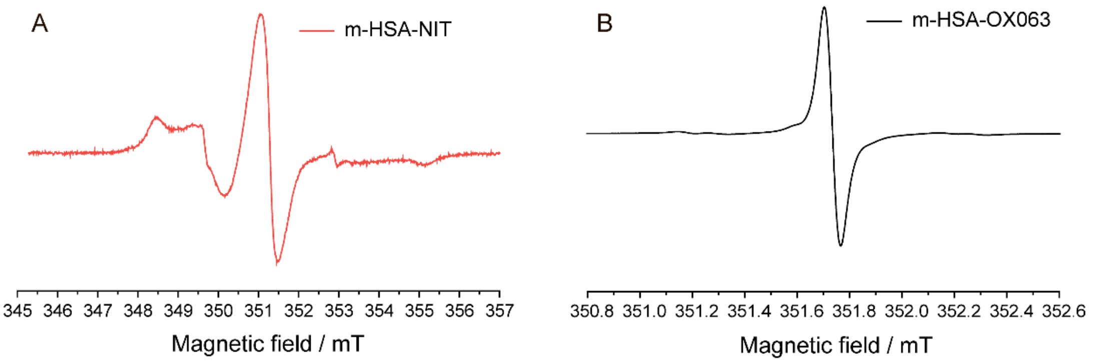

Figure S3. X-band first-derivative CW EPR spectra of m-HSA-NIT and m-HSA-OX063 samples measured at room temperature.

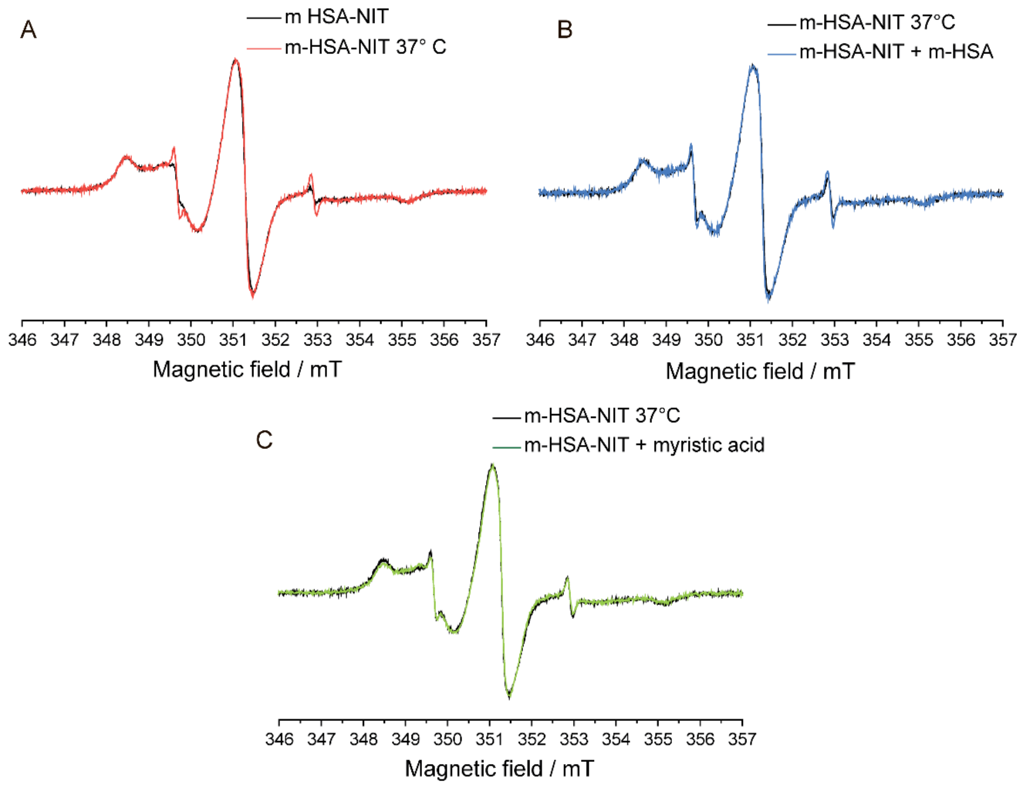

Figure S4. X-band first-derivative CW EPR spectra of m-HSA-NIT samples measured at room temperature. Samples were heated for 1 hour at 37 °C before EPR measurements to model the native environment. A –comparison of unheated m-HSA-NIT and m-HSA-NIT 37 °C samples; B –comparison of m-HSA-NIT 37 °C and m-HSA-NIT mixed 1:1 with m-HSA-SH; C –comparison of m-HSA-NIT 37 °C and m-HSA-NIT mixed with myristic acid.

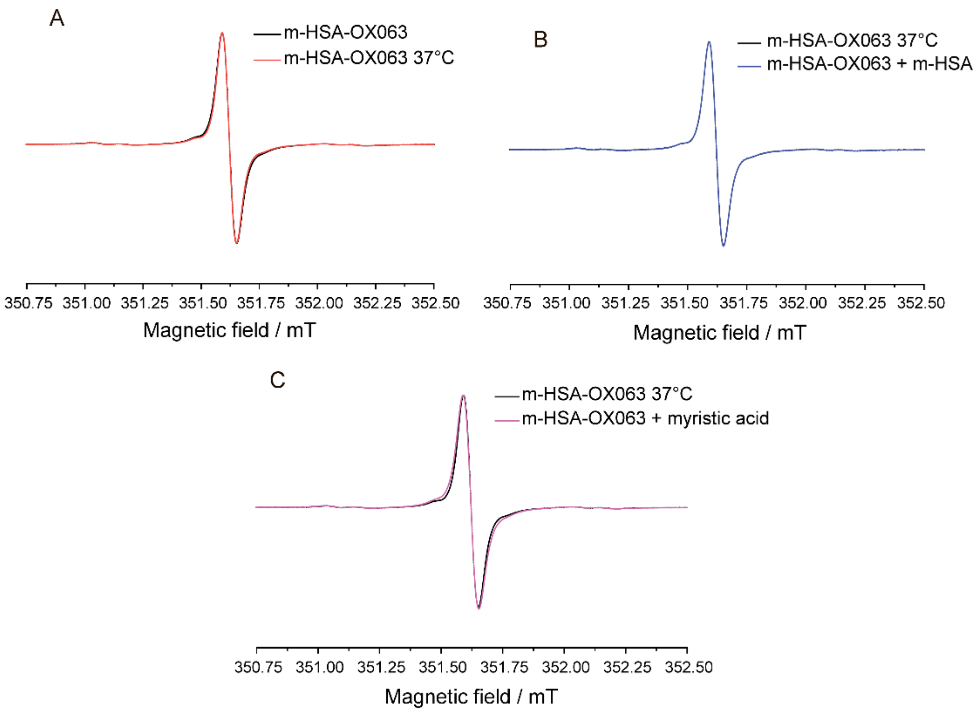

Figure S5. X-band first-derivative CW EPR spectra of m-HSA-OX063 samples measured at room temperature. Samples were heated for 1 hour at 37 °C before EPR measurements to model the native environment. A –comparison of unheated m-HSA-OX063 and m-HSA-OX063 37 °C samples; B – comparison of m-HSA-OX063 37 °C and m-HSA-OX063 mixed 1:1 with m-HSA-SH; C – comparison of m-HSA-OX063 37 °C and m-HSA-OX063 mixed with myristic acid.

Preparation of samples for DEER

Samples for DEER experiments were prepared in a 1:1 mixture of deuterated potassium PBS and the cryoprotectant glycerol-d8, placed in quartz EPR tubes and shock frozen. This procedure preserves the room temperature structure and conformation of biomacromolecules for DEER [7], ENDOR and pulsed EPR measurements. Reichenwallner, et al. [8] recently reported DEER experiments on nitroxide labeled HSA and found no evidence of dimers and no DEER signal. We see at least two significant differences between our experiments. First, they did not treat the sample with DTT to reduce Cys-34 to the thiol before labelling. As a result, roughly 2/3 of their HSA was unlabeled and statistically, only 1/9 of the dimers formed would have two labels to produce a DEER signal. In our samples, almost 100 % of the dimers were doubly labeled and gave a DEER signal. The lower amount of doubly labelled dimers drastically lowers their DEER amplitude changes relative to ours. Second, their sample buffer is largely a sodium phosphate buffer which is well-documented as being unable to control pH when a sample freezes because of differences in the solubility of the sodium salts during cooling [9–15]. Our samples were in potassium phosphate buffer which is free of this problem. We have verified that our DEER measurements were not reproducible in sodium phosphate buffer and therefore consistently use potassium PBS to preserve protein integrity whenever solutions are frozen.

DEER

DEER spectroscopy can provide the distance distribution between two or more spin labels in the range of 1.5 to 8 nm or more. Obtaining the distance distribution from DEER signal is an ill-posed mathematical problem since the same signal with only slight variations in noise can produce rather different distance distributions. To overcome this problem, some form of regularization is used, e.g., Tikhonov regularization. Recently, Worswick et al. [17] developed a new neural network method called DEERNet to solve this ill-posed problem. We found DEERNet to be consistently better for analyzing this DEER data than Tikhonov regularization and consequently DEERNet was used to obtain all the distance distributions.

The intensity change, or modulation depth, of the DEER signal depends on the percentage of spins in pairs relative to the total amount of spins in the sample. Thus, modulation depth analysis can quantifying the relative amount of spin labels in pairs, and can be used to characterize protein oligomers. Prior to analysis, the DEER signal must be separated from and background exponential decay, which is not possible using DEERNet, so Tikhonov regularization was used to obtain the background decay and the modulation depth, Figure S6 and S7. The background was modeled as an exponential function assuming a three-dimensional distribution of spins in the sample using the same start – 500 ns – and cutoff – 2000 ns parameters for all data sets. The modulation depth was then extracted, Table S2. The DEERNet and the Tikhonov regularization capabilities of DEERAnalysis2019 software package [18] were used in processing the DEER data. Raw DEER data sets are shown in Figure S8 and S9.

DEER experiments were recorded on a commercial X/Q-band Bruker Elexsys E580 spectrometer equipped with an Oxford flow helium cryostat and temperature control system. Samples were placed in quartz tubes (OD 1.7 mm, ID 1.1 mm), shock-frozen in liquid nitrogen and investigated at Q-band at temperatures of 50 K for nitroxide and 80 K for OX063 trityl. For DEER measurements a standard four-pulse sequence was used with a two-step phase cycle. Parameters for NIT (MTSL): π-pulse lengths of 20 ns for probe (υ_{probe}) and 18 ns for pump (υ_{pump}) frequency, time increment of the inversion pulse was 8 ns, values of τ_1 was 340 ns and τ_2 was 3500 ns. The pump pulse was applied near the maximum of the spectrum, and probe pulse was placed at the center of the spectrum. Thus, the measurements were done at a field position of 2.14 mT higher than the position of the pump pulse, and $\Delta\upsilon = (\upsilon_{pump} - \upsilon_{probe}) = 60$ MHz was

kept constant, Figure S10, A. Parameters for OX063: π-pulse lengths of 48 ns for probe (υ_{probe}) and 46 ns for pump (υ_{pump}) frequency, time increment for the inversion pulse position was 6 ns, the values of τ_1 and τ_2 were 340 ns and 3500 ns, respectively. The pump pulse was applied at the maximum of the spectrum, and probe pulse was placed at ^{13}C satellite EPR line. Hence, the measurements were made at a field position 0.71 mT lower than the maximum of the spectrum, and $\Delta\upsilon = (\upsilon_{pump} - \upsilon_{probe}) = -20$ MHz was kept constant, Figure S10, B.

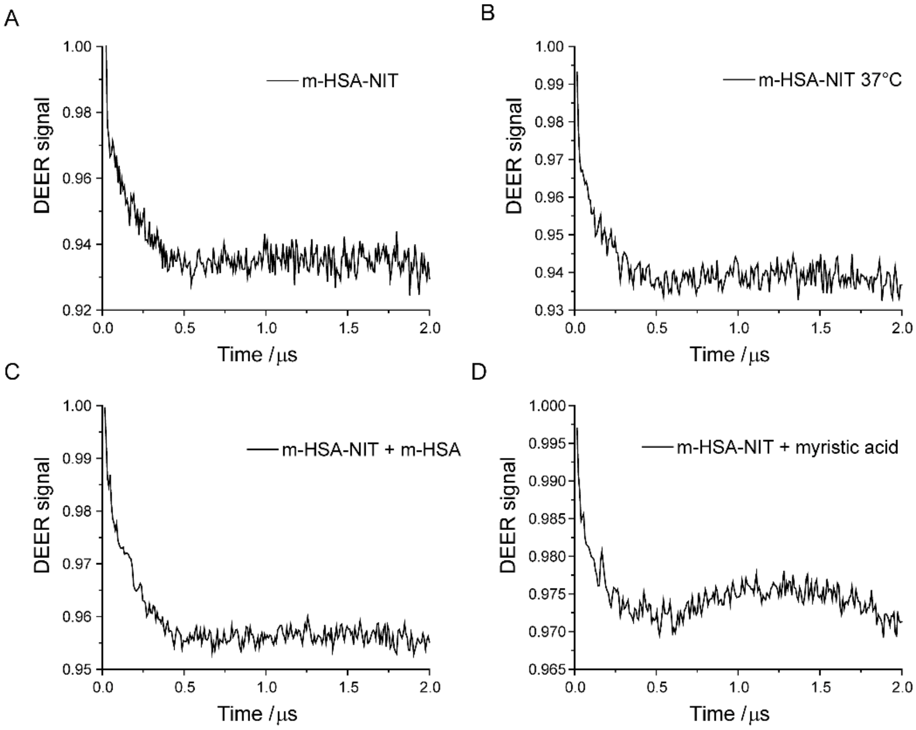

Figure S6. Background corrected DEER traces for m-HSA-NIT using Tikhonov regularization in DeerAnalysis2019. A –m-HSA-NIT; B - m-HSA-NIT heated at 37 °C; C - m-HSA-NIT mixed 1:1 with m-HSA; D - m-HSA-NIT with myristic acid.

Table S2. Modulation depth derived with Tikhonov regularization for spin labeled HSA. The modulation depths with all spins in pairs is 23% for nitroxides and 25% for OX063.

Spin label / Sample	HSA monomer	HSA covalent oligomers	Heated HSA monomer	HSA monomer + buffer	HSA monomer + unlabeled HSA	HSA monomer + myristic acid
Nitroxide	7%	9%	6,5%	6%	4.5%	2.5%
OX063	11%	13%	10%	12%	8%	5%

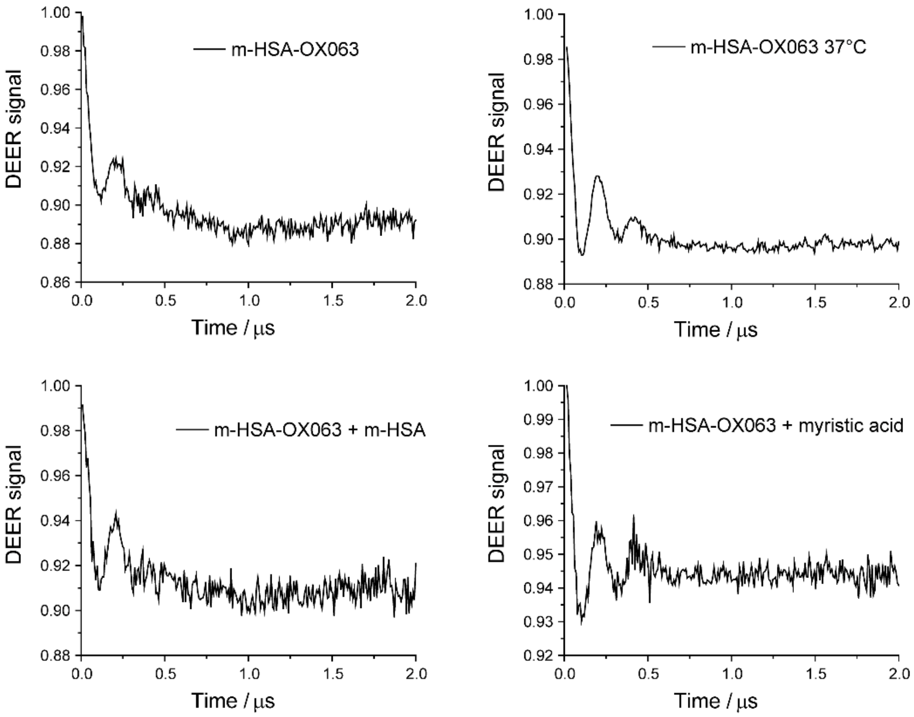

Figure S7. Background corrected DEER traces for HSA-OX063 using Tikhonov regularization in DeerAnalysis2019. A –m-HSA-NIT; B - m-HSA-OX063 heated at 37 °C; C - m-HSA-OX063 mixed 1:1 with m-HSA; D - m-HSA-OX063 with myristic acid.

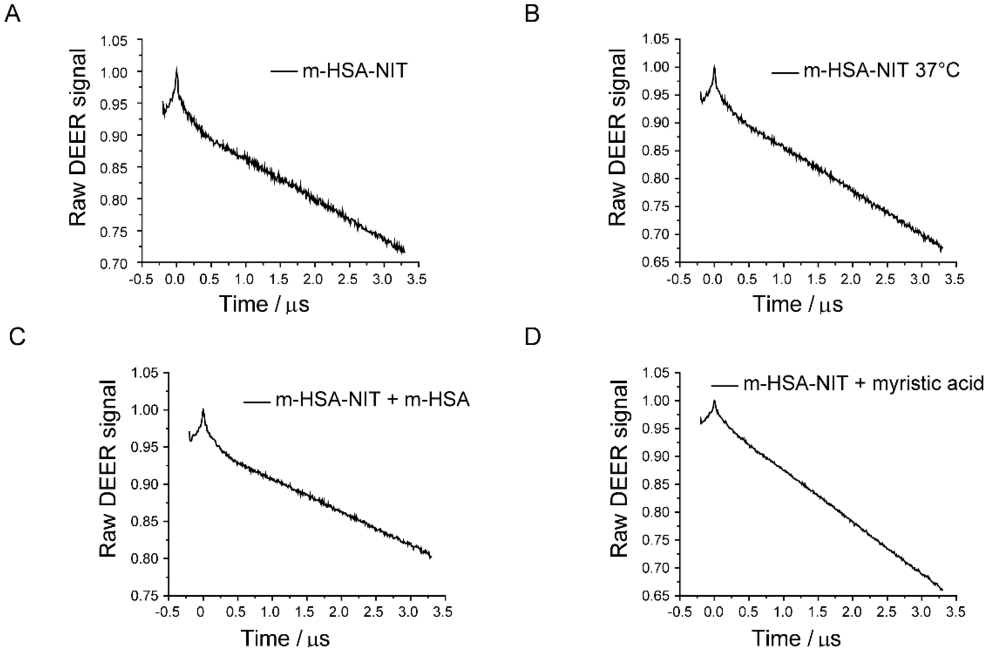

Figure S8. Raw Q-band DEER data at 50 K for A – m-HSA-NIT; B –m-HSA-NIT heated at 37 °C; C – m-HSA-NIT mixed 1:1 with m-HSA; D – m-HSA-NIT with myristic acid.

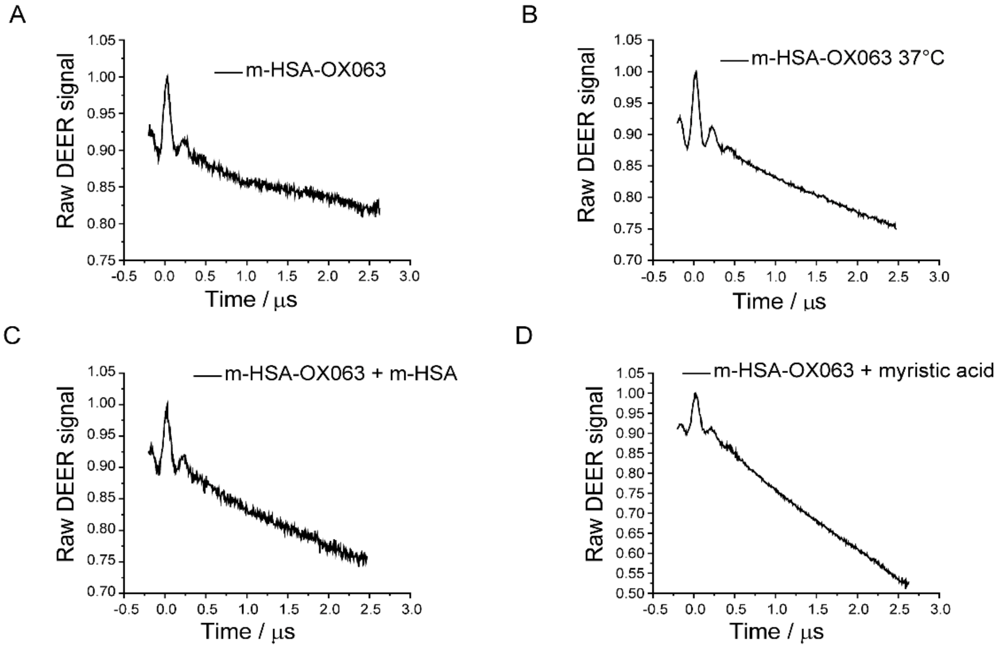

Figure S9. Raw Q-band DEER data at 80 K for A – m-HSA-OX063; B –m-HSA-OX063 heated at 37 °C; C – m-HSA-OX063 mixed 1:1 with m-HSA; D – m-HSA-OX063 with myristic acid.

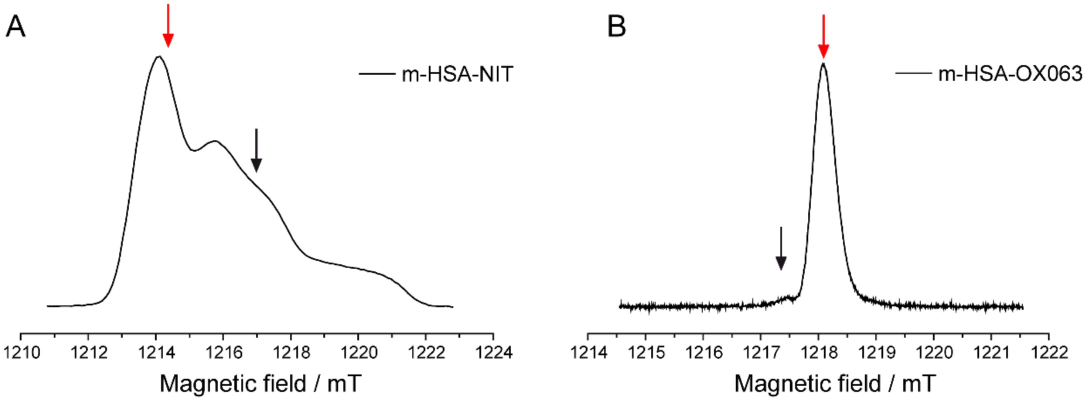

Figure S10. A - echo detected Q-band spectra for m-HSA-NIT sample at 50 K; B - echo detected Q-band spectra for m-HSA-OX063 sample at 50 K. Red arrows show positions of the pump pulses, and black arrows – positions of the probe pulses.

Characterization of HSA conjugates Structures by Dynamic light scattering (DLS)

Dynamic light scattering (DLS) was used to monitor the changes in HSA size and aggregation in the HSA conjugate samples. The size distributions of the protein samples were determined by DLS using a Zetasizer Nano-ZS (Malvern Panalytical Ltd., Malvern, UK) at 25 °C. The protein samples (5 μM) were prepared in PBS buffer and measurements of the size were conducted. DLS is more sensitive to protein aggregation and polydispersity than to small conformational changes. The basic distribution obtained from DLS measurement is intensity – all other distributions can be generated

from it. The intensity mode is suitable for investigating the aggregation process. It is sensitive to changes in the quantity of large particles. This is because large particles scatter much more light than small particles. The scattering intensity of a particle is proportional to the sixth power of its diameter from the Rayleigh approximation.

HSA solutions always contain some aggregated material, but the area, position, and the number of peaks of HSA aggregates are different (Figure S11-S14). In PBS solution m-HSA can form some aggregated material, while for m-HSA and m-HSA-SH there are no significant changes, Figure S11.

The effects of myristic acid on protein oligomer formation are presented in Figures S14-15. The addition of myristic acid to the samples of m-HSA-NIT and m-HSA-OX063 leads to a decrease of large diameter particles in the solution. This confirms that the fatty acid induces a well-defined conformation, different from the native one. It is known [19] that fatty acids mainly stabilize subdomains IA and IIIB. Also, the presence of ligands in subdomain IIIB strongly stabilizes that region. This suggests that binding with myristic acid will contribute to the transition in the I and IIIB domains and will affect oligomer formation. The data is consistent with the hypothesis of reversible albumin dimer formation by the I and III domains of the HSA obtained by PELDOR data.

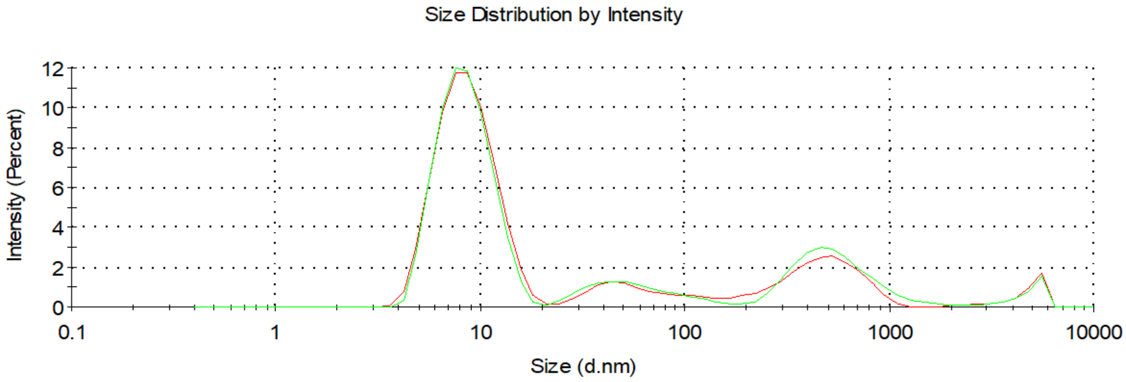

Figure S11. DLS size distribution of 5 μM m-HSA (green) and m-HSA-SH (red).

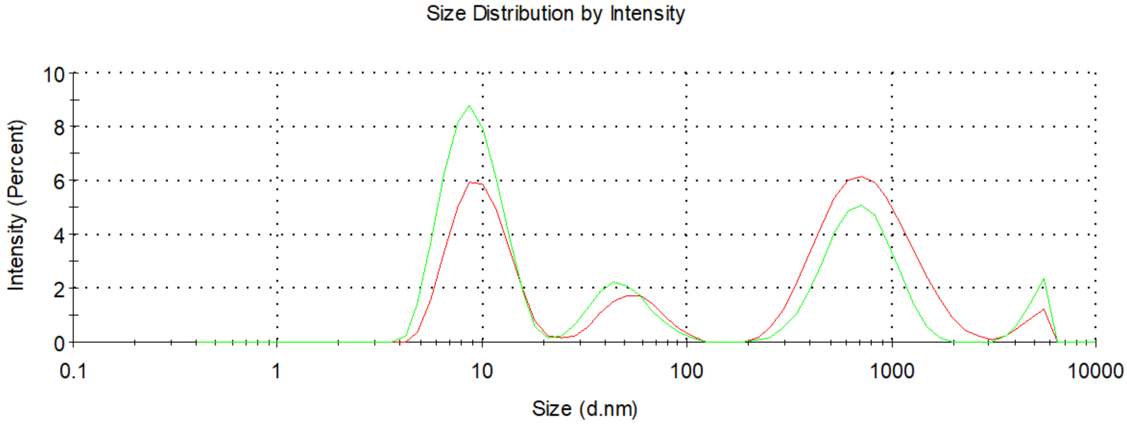

Figure S12. DLS size distribution of 5 μM m-HSA-NIT (green) and m-HSA-OX063 (red).

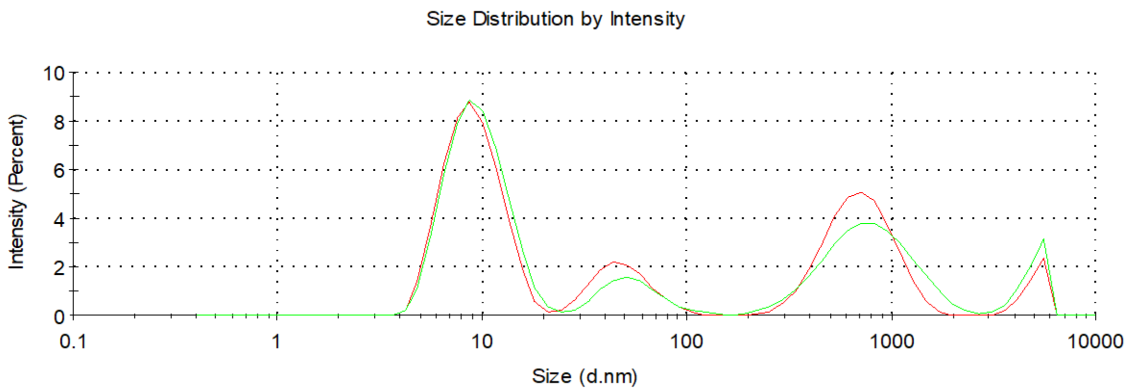

Figure S13. DLS size distribution of 5 µM m-HSA-NIT (red) and m-HSA-NIT titrated with myristic acid (green).

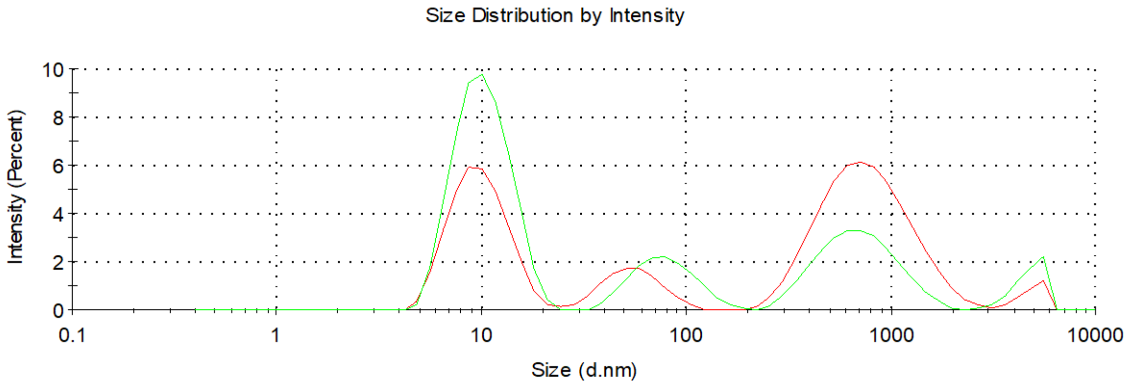

Figure S14. DLS size distribution of 5 µM m-HSA-OX063 (red) and m-HSA-OX063 titrated with myristic acid (green).

3. References

1. Janatova, J.; Fuller, J.K.; Hunter, M.J. The heterogeneity of bovine albumin with respect to sulfhydryl and dimer content. *J. Biol. Chem.* **1968**, *243*, 3612–3622.
2. Perczel, A.; Hollósi, M.; Tusnády, G.; Fasman, G.D. Convex constraint analysis: a natural deconvolution of circular dichroism curves of proteins. *Protein Eng.* **1991**, *4*, 669–679.
3. Krumkacheva, O.A.; Timofeev, I.O.; Politanskaya, L. V.; Polienko, Y.F.; Tretyakov, E. V.; Rogozhnikova, O.Y.; Trukhin, D. V.; Tormyshev, V.M.; Chubarov, A.S.; Bagryanskaya, E.G.; et al. Triplet Fullerenes as Prospective Spin Labels for Nanoscale Distance Measurements by Pulsed Dipolar EPR. *Angew. Chemie Int. Ed.* **2019**, *58*, 13271–13275, doi:10.1002/anie.201904152.
4. Tormyshev, I.; Chubarov, A.; Krumkacheva, O.; Trukhin, D.; Rogozhnikova, O.; Spitsina, A.; Kuzhelev, A.; Koval, V.; Fedin, M.; Bowman, M.; et al. A Methanethiosulfonate Derivative of OX063 Trityl: A Promising and Efficient Reagent for SDSL of Proteins. *Chem. - A Eur. J.* **2020**, *26*, 1–9, doi:10.1002/chem.201904587.
5. Funk, W.E.; Li, H.; Iavarone, A.T.; Williams, E.R.; Riby, J.; Rappaport, S.M. Enrichment of cysteinyl adducts of human serum albumin. *Anal. Biochem.* **2010**, *400*, 61–68, doi:10.1016/j.ab.2010.01.006.
6. Tormyshev, V.M.; Chubarov, A.S.; Krumkacheva, O.A.; Trukhin, D. V.; Rogozhnikova, O.Y.; Spitsyna, A.S.; Kuzhelev, A.A.; Koval, V. V.; Fedin, M. V.; Godovikova, T.S.; et al. Methanethiosulfonate Derivative of OX063 Trityl: A Promising and Efficient Reagent for Side-Directed Spin Labeling of Proteins. *Chem. – A Eur. J.* **2020**, *26*, 2705–2712, doi:10.1002/chem.201904587.
7. Tsvetkov, Y.D.; Bowman, M.K.; Grishin, Y.A. *Pulsed Electron–Electron Double Resonance: Nanoscale Distance Measurement in the Biological, Materials and Chemical Sciences*; Springer International Publishing, 2019;
8. Reichenwallner, J.; Hauenschild, T.; Schmelzer, C.E.H.; Hülsmann, M.; Godt, A.; Hinderberger, D. Fatty Acid Triangulation in Albumins Using a Landmark Spin Label. *Isr. J. Chem.* **2019**, *59*, 1059–1074, doi:10.1002/ijch.201900073.
9. Astl, G.; Mayer, E. Alkali cation effect on carbonyl-hemoglobin's and -myoglobin's conformer populations when exposed to freeze-concentration of their phosphate-buffered aqueous solutions. *Biochim. Biophys. Acta (BBA)/Protein Struct. Mol.* **1991**, *1080*, 155–159, doi:10.1016/0167-4838(91)90143-N.
10. Gomez, G.; Pikal, M.J.; Rodriquez-Hornedo, N. Effect of Initial Buffer Composition on pH Changes During Far-

From-Equilibrium Freezing of Sodium Phosphate Buffer Solutions Gerardo. *Pharm. Res.* **2001**, *18*, 90–97.
11. Kolhe, P.; Amend, E.; Singh, S.K. Impact of freezing on pH of buffered solutions and consequences for monoclonal antibody aggregation. *Biotechnol. Prog.* **2010**, *26*, 727–733, doi:10.1002/btpr.377.
12. Mezhebovsky, T.; Routhier, E.; Sass, P.; Shahrokh, Z. Enabling Freeze-Thaw Stability of PBS-Based Formulations of a Monoclonal Antibody Part I: Freeze-Thaw Stress Testing. *Biopharm Int* **2016**, *29*, 33–39.
13. Pikal-Cleland, K.A.; Rodríguez-Hornedo, N.; Amidon, G.L.; Carpenter, J.F. Protein denaturation during freezing and thawing in phosphate buffer systems: Monomeric and tetrameric β-galactosidase. *Arch. Biochem. Biophys.* **2000**, *384*, 398–406, doi:10.1006/abbi.2000.2088.
14. Thomas, L.; Mezhebovsky, T.; Routhier, E.; Sass, P.; Shahrokh, Z. Enabling Freeze-Thaw Stability of PBS-Based Formulations of a Monoclonal Antibody. *Biopharm Int* **2016**, *29*, 31–36.
15. Thorat, A.A.; Suryanarayanan, R. Characterization of Phosphate Buffered Saline (PBS) in Frozen State and after Freeze-Drying. *Pharm. Res.* **2019**, *36*, 98, doi:10.1007/s11095-019-2619-2.
16. Tsvetkov, Y.D.; Bowman, M.K.; Grishin, Y.A. *Pulsed Electron–Electron Double Resonance*; Springer International Publishing: Cham, 2019; ISBN 978-3-030-05371-0.
17. Worswick, S.G.; Spencer, J.A.; Jeschke, G.; Kuprov, I. Deep neural network processing of DEER data. *Sci. Adv.* **2018**, *4*, eaat5218, doi:10.1126/sciadv.aat5218.
18. Jeschke, G.; Chechik, V.; Ionita, P.; Godt, A.; Zimmermann, H.; Banham, J.; Timmel, C.R.; Hilger, D.; Jung, H. DeerAnalysis2006—a comprehensive software package for analyzing pulsed ELDOR data. *Appl. Magn. Reson.* **2006**, *30*, 473–498, doi:10.1007/BF03166213.
19. Guizado, T.R.C. Analysis of the structure and dynamics of human serum albumin. *J. Mol. Model.* **2014**, *20*, doi:10.1007/s00894-014-2450-y.
